# The role of employee surveys to promote physical health and healthy lifestyles at the workplace: a scoping review

**DOI:** 10.1186/s12889-025-24716-7

**Published:** 2025-09-24

**Authors:** Arvid Heikinniemi, Sebastian Heikkilä, Oskar Halling Ullberg, Britt Hallingberg, Katarina Bälter

**Affiliations:** 1 School of Health, Care and Social Welfare, Mälardalen University, Universitetsplan 1, Västerås, 72123 Sweden; 2 Centre for Health, Activity and Wellbeing Research, Cardiff School of Sport and Health Sciences, Cardiff Metropolitan University, Cardiff, UK; 3 Department of Medical Epidemiology and Biostatistics, Karolinska Institute, Stockholm, Sweden

**Keywords:** Scoping review, Employee surveys, Lifestyle, Health, Public health, Health promotion, Occupational safety, Organizational development

## Abstract

**Background:**

Employee surveys aim to assess employees’ attitudes and work environments and offer a strategic approach to workplace improvement. However, in these surveys, areas related to health and lifestyle are often overlooked, despite their relevance to the wellbeing and performance of the employees. The aim of this scoping review was to examine the role of employee surveys in promoting physical health and healthy lifestyles among employees at the workplace.

**Methods:**

To be eligible for inclusion, published articles needed to investigate employees’ physical health or lifestyles and utilize employee survey data, be published in English within the last 10 years (2014–2024) and be available in full text in the databases ProQuest one business, Emerald Insight, PubMed, Web of Science and Scopus.

**Results:**

1,550 studies were screened, and eight studies fulfilled the inclusion criteria. Of these, two studies assessed data at two time points to study change over time, and only one study aimed to influence behavior change of employees. This demonstrates a lack of evidence-based methods for linking employee surveys data to health promoting initiatives in a workplace context.

**Conclusions:**

This scoping review highlights an urgent knowledge gap in the literature. Future research should explore the process of using employee surveys for identifying health related problems, designing and implementing solutions, follow-up improvements, and thereby creating a workplace health promoting loop process.

**Registration:**

A study protocol has been preregistered on Open Science Framework with registration number xza4m.

**Supplementary Information:**

The online version contains supplementary material available at 10.1186/s12889-025-24716-7.

## Background

Employee surveys aims to assess employees’ attitudes and work environment, serving as a strategic approach to workplace improvement [1–3]. Typical areas that these surveys cover are leadership [4, 5], workplace environment and safety [1, 6], work-related risks [1, 7], employee engagement [1, 8] and turnover intent [1].

However, areas such as health and lifestyle are often overlooked in employee surveys, despite their relevance to the organizations’ most valuable resource: human capital. A healthy lifestyle including healthy food habits, physical activity, limiting intake of alcohol and tobacco use, time for recovery and rest are primary preventive measures for poor health outcomes such as central obesity, type II diabetes, high blood pressure and elevated blood lipids [9]. Since these health outcomes are linked to reduced productivity, financial strain, and increased absenteeism [10–12] there is an urgent need to integrate health promotion into systematic organizational development. However, current health-related topics in many employee surveys focus solely on occupational risk factors and workplace safety [1], and not on physical health or healthy lifestyles. Although, there are some exceptions. For instance, the CPH-NEW All Employee Survey [13] includes questions about general health, fruit and vegetable consumption, physical activity and pain. Additionally, the OSA-Survey (Organizational and Social Work Environment) comprises questions on recovery and psychological safety [14]. But organizations often rely on external consultancies for conducting employee surveys, using pre-defined questions [15, 16], where areas such as physical health and healthy lifestyles are mostly overlooked.

According to the recently launched directive on Corporate Sustainability Reporting [17], it will be mandatory for companies in the European Union to produce a sustainability report, including an in-depth description of the organization’s impact on human and planetary health. Thus, the employee survey has the potential to play a key role in assessing health-related factors, monitoring appropriate health promoting activities and evaluating their effect on employees, and make this information available for investors and stakeholders in the yearly sustainability report.

To the best of our knowledge, there is no literature review evaluating the linkage of employee survey data to employee health, beyond workplace risk factors and safety. This scoping review therefore aims to study the role of employee surveys in promoting physical health and healthy lifestyles among employees at the workplace. This may identify current knowledge gaps and guide future research in bringing occupational safety, employee health and lifestyles, workplace health promotion initiatives and organizational development closer together.

## Methods

This scoping review investigates and describes the published up-to-date data on the role of employee surveys to promote and follow-up health and healthy lifestyle among employees. It complies with the Preferred Reporting Items for Systematic Reviews and Meta-Analysis, Extension for Scoping Reviews (PRISMA-ScR) [18] and the methodology of H Arksey and L O’Malley [19]. The aim was constructed with the Population, Concept and Context (PCC) mnemonic [20], where Population corresponded to employees at the workplace, Concept to the physical health and healthy lifestyle and the Context to employee surveys. The aim of the review was slightly changed as compared to the preregistered protocol to better capture the scope and aim of the review, see Open Science Framework February 19, 2025, with registration number xza4m [21].

### Eligibility criteria

To be deemed eligible for inclusion, published research articles needed to investigate employees’ physical health or lifestyles, such as overweight/obesity, metabolic health, stress, sleeping habits, musculoskeletal pain, mild psychological symptoms, exercise, diet, outdoor experiences or recovery. Additionally, the eligible articles must have utilized employee survey data initiated and performed by or at an organization/company, or by an external consultancy at a specific organization/company. The study participants consisted of employees from the respective organization/company. The eligibility criteria are explained in more detail in Table 1.

**Table 1:** Eligibility criteria

	Inclusion	Exclusion
Population – Employees at the workplace	Articles that investigate employees at the workplace, regardless of role or type of business.	Articles that investigate populations other than employees.
Concept – Physical health and healthy lifestyle	Articles that investigate health or lifestyle-related outcomes, such as overweight or obesity, metabolic health, stress, sleeping habits, musculoskeletal pain, exercise, diet, outdoor experiences, recovery or mild psychological symptoms.	Articles that investigate psychiatric or clinical diagnoses or more severe psychological symptoms, such as depression, cancer or burnout. Articles that only investigate work-related outcomes, such as engagement or job demands.
Context – Employee surveys	Articles that utilize employee survey data initiated and performed by or at an organization/company, or by an external consultancy at a specific organization/company.	Articles that utilize large-scale population data that cannot be derived from any specific workplace.

The review included all kinds of business, companies, and organizations but to ensure a baseline of quality and time relevance, only peer reviewed empirical articles published within the last 10 years (2014–2024) were included. However, following the methodology by H Arksey and L O’Malley [19], this scoping review did not assess the quality of included articles, nor did it include gray literature, such as conference papers and commentaries. All eligible articles must be published in English and available in full text.

### Information sources and search strategy

The search strategy was developed with the help of librarians at Mälardalen University and contained three steps. The aim was first discussed with the librarians to determine relevant databases and search terms. Additionally, several other search terms were identified by the librarians and validated by the first and last authors. Finally, pilot tests of the research strategy were performed in Web of Science and Medline before it was finalized and delivered on November 28, 2024. The databases used included ProQuest One Business, Emerald Insight, PubMed, Web of Science and Scopus. The search strategy was examined using the PRESS checklist [22]. Table 2 contains the complete search strategy employed in PubMed.

**Table 2:** Search strategy in PubMed

1. [Words for employee survey]	“employee* survey*“[Title/Abstract] OR “employment survey*“[Title/Abstract] OR “employer survey*“[Title/Abstract] OR “employee* question*“[Title/Abstract] OR “employment question*“[Title/Abstract] OR “workforce survey*“[Title/Abstract] OR “workforce question*“[Title/Abstract] OR “organization* survey*“[Title/Abstract] OR “organization* question*“[Title/Abstract] OR “organisation* survey*“[Title/Abstract] OR “organisation* question*“[Title/Abstract] OR “worksite survey*“[Title/Abstract] OR “worksite measure*“[Title/Abstract]
2.	“employer questionnaire“[tiab:~0]
3.	“worksite questionnaire“[tiab:~0]
4.	1 OR 2 OR 3
5. [Words for *health*]	health*[Title/Abstract] OR wellness[Title/Abstract] OR well-being[Title/Abstract] OR stress[Title/Abstract] OR “physical activit*“[Title/Abstract] OR exercise[Title/Abstract] OR sport*[Title/Abstract] OR sedentary[Title/Abstract] OR “physical inactivity“[Title/Abstract] OR diet*[Title/Abstract] OR eat[Title/Abstract] OR eating[Title/Abstract] OR food*[Title/Abstract] OR nutrition*[Title/Abstract] OR obesity[Title/Abstract] OR obese[Title/Abstract] OR overweight*[Title/Abstract] OR sleep*[Title/Abstract]
6. [Corresponding MeSH]	“health promotion“[MeSH Terms] OR “health behavior“[MeSH Terms] OR “healthy lifestyle“[MeSH Terms] OR “occupational health“[MeSH Terms] OR “physical fitness“[MeSH Terms] OR “exercise“[MeSH Terms] OR “sports“[MeSH Terms] OR “sedentary behavior“[MeSH Terms] OR “diet“[MeSH Terms] OR “diet, healthy“[MeSH Terms] OR “obesity“[MeSH Terms] OR “overweight“[MeSH Terms] OR “sleep“[MeSH Terms] OR “occupational stress“[MeSH Terms]
7.	5 OR 6
8.	4 AND 7
Limitations	Publication date: 2014–2024

Some exceptions were made in the search strategy. Firstly, in PubMed, two phrases (“employer question*” and “worksite question*”) were not included in PubMed’s phrase index. These phrases were instead searched using a proximity search [23]. Secondly, only 500 articles were extracted from Emerald Insight due to the limitation on the number of articles that could be extracted in a single search.

### Screening

**Fig. 1:**
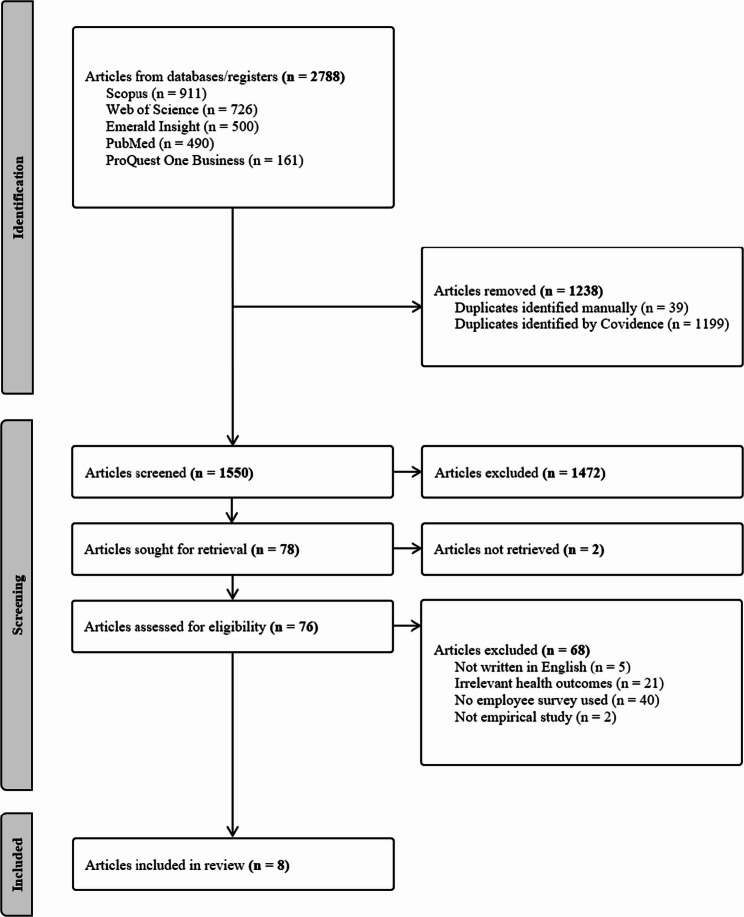
PRISMA flow diagram of the screening process

The screening process was handled using Covidence to manage research articles and remove duplicates. Two reviewers (AH & SH) were responsible for screening the articles and assessing relevance based on the eligibility criteria. The first 10 articles were used as a pilot. For these 10 articles and the rest in the dataset, titles and abstracts were screened and compared between the reviewers to ensure that both understood the eligibility criteria. Disagreements of inclusion among any articles were discussed between the reviewers and, if necessary, a third co-author. Once all titles and abstracts were screened, the reviewers continued to screen the full texts of potentially eligible articles. Key sections of the articles were screened, mainly methods and results, to see if the study achieved the eligibility requirements and could answer the aim. Any disagreements in this stage were solved as in the previous phase, with discussions between the reviewers and sometimes a third co-author. The reference lists of all eligible articles were also screened to search for relevant studies, and the same eligibility criteria were applied. The full screening process is visualized using a PRISMA flow diagram, see Fig. 1.

### Data charting

Key information relevant to the review was charted from the eligible articles as described by H Arksey and L O’Malley [19]. A data charting form was formulated by the first author to ease the charting of data. After reviewing the form with the last author, it was decided to collect the following data:

- General information: Authors, year, title, aim, study design, country, sample and sample size, demographics of participants (age, gender), setting.
- Health, lifestyle & wellbeing: Investigated health aspects, lifestyles or behaviors, measures, investigated associations.
- Employee surveys: Description of the employee survey, the role of the employee survey, the role of the organization.

Two items were changed from the pre-registered protocol. “line of business” was changed to “occupational setting” and “expected impact of the study” was changed to “investigated associations”.

The same reviewers involved in the screening process also charted the data. The first two eligible articles were part of a pilot round to test the data charting form to make sure both reviewers captured the same information. The reviewers had recurrent meetings to discuss the data charted from the articles to make sure that no relevant information was missed. If any data were unclear, the reviewers would first contact the other co-authors of the scoping review to discuss the material, and if necessary, an attempt was made to contact the corresponding author of the articles. However, no such contact was necessary for this study. Any missing information in the studies were coded with “N/A” (Not available). Microsoft Excel was used to store all the charted data.

## Results

In total, 1,550 articles were screened based on titles and abstracts, and of these, 78 articles were identified for full text review. Among these, in total 70 were excluded since the articles was not based on an employee survey (*n* = 40), focused on irrelevant health-related outcomes (*n* = 21), were not written in English (*n* = 5), could not be retrieved (*n* = 2), or was not an empirical study (*n* = 2). The full screening process resulted in eight published articles that fulfilled the inclusion criteria, illustrated in Fig. 1. The reference lists of these included articles were screened, but no additional articles that fulfilled the inclusion criteria were identified. All relevant data were charted according to the data charting form and are presented in additional file 1.

###  Description of studies

Each article described one unique study, and the final set of studies originated from seven different countries: Germany (*n* = 2), the USA (*n* = 1), Denmark (*n* = 1), Australia (*n* = 1), Sweden (*n* = 1), Switzerland (*n* = 1) and Iran (*n* = 1). Study designs were cross-sectional (*n* = 5), quasi-experimental (*n* = 1), longitudinal (*n* = 1) and an interview study (*n* = 1). Study participants ranged from 127 [24] to 86,257 respondents [25]. All studies utilized employees from different occupational settings as participants, whereas the study by R Larsson, I Åkerlind and H Sandmark [26] only included managers. The age of the participants ranged from 15 to 60 + years, with the majority being between 31 and 60 years old [24, 27, 28]. Ages were not reported in three studies [25, 29, 30] while two studies reported mean age of 35 [31] and 54 years [26], respectively. In four studies, the majority of the participants were males [24, 27, 28, 31], while the remaining three studies did not report the participants’ gender [25, 29, 30].

Theories and models are used sparsely for designing or interpreting the results in these studies although, one study [24], used the Job Demands-Resources model as a framework to investigate work characteristics and psychological wellbeing. Traces of other models and theories were present in some studies, as single or multiple questions in the survey, for example The Effort-Reward Imbalance Model [30] and Self-Efficacy [25], but not used systematically as frameworks throughout the whole process.

### Employee surveys

Seven of the studies were based on quantitative data and for the purpose of this review, work-related factors captured by employee surveys were considered as exposure while health and healthy lifestyle were considered the outcome. Most studies (*n* = 6) used employee surveys to collect data about the association between work-related factors and health.

Five of the eight studies were based on data from one assessment in cross-sectional studies [27–31], whereas two studies assessed data at two time points: pre- and post-assessments during an intervention targeting physical inactivity [25] as well as assessments of change in transformational leadership and psychological wellbeing [24]. However, only one of these studies linked data between the two time points [24]. One study was based on qualitative data, via interviews with managers working with workplace health promotion [26].

The aim and process of using employee surveys varied. Two studies reported that the employee survey process was designed by the employer or a consultancy [29, 30]. Additionally, some studies described the aim of the survey as a tool to manage the workplace [26, 27] or to evaluate and identify workplace risks [24]. One study used a separate wellness survey (not named) parallel to the employee survey [25]. Meanwhile, two studies did not report the characteristics of the employee survey process or its aim [28, 31].

### Health outcomes

The type of health and lifestyle aspects that were investigated varied, while in four studies focused on outcomes related to physical health. These included *physical activity/inactivity* (*n* = 2), Body Mass Index (*BMI)* (*n =* 2), *musculoskeletal symptoms* (*n* = 2), *general physical health* (*n = 1)*, *smoking* (*n = 1)*, *fruit and vegetable consumption* (*n = 1)*, and *alcohol consumption* (*n* = 1). Five studies measured psychological health including *psychological distress or wellbeing (n* = 4*) psychosocial factors (n* = 1*)*, *stress* (*n* = 1) and *burnout* (*n* = 1). Social health was measured in four studies and included *social support* (*n* = 2) and *social capital* (*n* = 2). In the interview study by R Larsson, I Åkerlind and H Sandmark [26], managers focused on implementing fitness programs that promoted physical activity and healthy lifestyles at the workplace. While the programs were reviewed positively, employees criticized them for relying too heavily on the fitness outcomes [26].

How health and lifestyle was assessed varied between the studies and two studies assessed general health using a single question regarding the employees’ self-rated health, though the way the question was framed was not reported [28, 29]. One study assessed general physical health among employees using the SF-12 Physical Component Scale [30]. Two studies measured physical activity; L Jarman, A Martin, A Venn, P Otahal, R Taylor, B Teale and K Sanderson [30] used items from the Long International Physical Activity Questionnaire whereas TM Schult, SK Schmunk and ER Awosika [25] used one single question phrased like “During the past month, other than your regular job, did you participate in any physical activities or exercises such as running, calisthenics, golf, gardening, or walking for exercise?”. BMI was calculated using self-reported information about height and weight in two studies [30, 31]. L Jarman, A Martin, A Venn, P Otahal, R Taylor, B Teale and K Sanderson [30] also assessed smoking habits, as well as consumption of fruits, vegetable, and alcohol, but there was no information about how the questions were formulated. Furthermore, musculoskeletal symptoms in the back, shoulder, or neck were assessed in two studies, using either the Nordic Musculoskeletal Questionnaire [31] or an unspecified questionnaire from the Swiss Health Survey [28]. All items by L Jarman, A Martin, A Venn, P Otahal, R Taylor, B Teale and K Sanderson [30], except for psychological distress, were assessed using a research based questionnaire, and not an employee survey.

L Jarman, A Martin, A Venn, P Otahal, R Taylor, B Teale and K Sanderson [30] assessed psychological distress among employees with the Kessler-10 scale, which includes questions on tiredness, nervousness, hopelessness, and more [32]. Another study estimated stress with similar indicators, but with a single question from the Occupational Stress Questionnaire [28]. Other measures of psychological distress were the COPSOQ II Questionnaire, applied by A-K Loekke [29], with questions such as: “How often have you felt worn out, been emotionally exhausted, been irritable, and been stressed?”. O Hämmig [28] used the Copenhagen Burnout Inventory with questions such as: “How often do you feel emotionally drained?” and “How often do you feel empty and exhausted at the end of the day?”. Two studies used the WHO-5 scale to measure psychological wellbeing, with questions such as “Over the last two weeks I have felt cheerful and in good spirits” [24, 27]. Lastly, one study used the Effort Reward Imbalance questionnaire to assess psychosocial factors among employees [30].

Two studies measured social support, one with an unspecified scale for perceived social support, using 12 items from a Swiss stress study [28], and one with five unspecified items including social support from colleagues and supervisors [29]. Additionally, social capital was assessed in two studies: A-K Loekke [29] used an altered Social Capital Scale with questions such as “Is your work recognized and appreciated by the management?” and “Do the employees in general trust each other?”, while L Lindert, S Zeike, K-EA Choi and H Pfaff [24] used the SOCAPO-E scale, which covered mutual understanding, trust, teamwork, mutual help, and shared values.

### Associations between work-related factors and health

As the eight studies included in the present scoping review were considerably heterogenous with respect to aim, study design, how data were collected and how many times data were collected per person, how questions were formulated, type of company or organization, number of employees and country, but the main findings are usually reported as associations between work-related factors and health outcomes. P Piranveyseh, M Motamedzade, K Osatuke, I Mohammadfam, A Moghimbeigi, A Soltanzadeh and H Mohammadi [31] reported associations where higher levels of organizational outcomes, including organizational climate, computer work, work-family balance, job control, educational level, perception of leadership, job-related resources, quality of customer service, co-worker support, rewards, cooperation, BMI and support for development were associated with higher levels of musculoskeletal disorders, although in different parts of the body. Only the study by TM Schult, SK Schmunk and ER Awosika [25] used the employee survey to evaluate a health promoting initiative. The initiative reduced the level of physical inactivity from 25,3% to 16,1% among Veterans Health Administration employees. Other outcomes indicated that the initiative had overall positive effects, reporting that employees’ were more physically active, had a greater number of days of perceived good health, and had increased options for physical activity, such as bike racks or on-site fitness centers [25].

The other six studies mainly include measures of psychological and social outcomes. L Jarman, A Martin, A Venn, P Otahal, R Taylor, B Teale and K Sanderson [30] investigated psychological distress and, using logistic modelling, identified younger age, unmarried status, state service tenure, higher BMI, high risk alcohol consumption, daily smoking and upper tertiles of effort reward imbalance as the strongest predictors of high prevalence of psychological distress. However, other variables were also contributing predictors of high psychological distress. Additionally, the authors of the same study reported that the employee survey appeared to overestimate the prevalence of psychological distress, when compared to the researcher-employed survey applied during the following year in the same study and population (although using different sampling methods) [30]. In the study by L Lindert, S Zeike, K-EA Choi and H Pfaff [24], employees’ psychological wellbeing did not predict employees’ perceptions of transformational leadership over time, nor was the reverse association identified. However, male employees perceived higher levels of transformational leadership, and employees’ with higher social capital experienced higher levels of psychological wellbeing [24]. Psychological wellbeing was also investigated in the study by L Lindert, K-EA Choi, H Pfaff and S Zeike [27]. In the study, high levels of individual and organizational health literacy, and health supporting leadership, all had an individual association with high psychological wellbeing among employees. Furthermore, organizational health literacy and health supporting leadership both partially mediated the relationship between individual health literacy and psychological wellbeing [27]. In the study by A-K Loekke [29], higher levels of social capital was associated with lower levels of poor self-reported general health and psychological distress, and higher levels of job satisfaction and workplace commitment. O Hämmig [28] reported that limited sources of social support (0–2 sources) were highly associated with poor self-rated health, more musculoskeletal symptoms, and higher feelings of stress and burnout, as opposed to many sources of social support (5–6 sources). Additionally, limited sources of social support were also highly associated with feelings of being overwhelmed at work, having difficulties switching off after work, low job satisfaction and higher turnover intentions. Among all sources of social support, direct supervisors emerged as most important source for both health and work-related outcomes, meaning that having social support from a direct supervisor protected employees from health-related and work-related problems [28].

In the study based on interviews with managers working with workplace health promotion [26], the manager followed a problem-solving cycle, in which the employee survey allowed managers to evaluate local demands related to employee health and develop strategies to address health-related challenges. However, managers reported that the employee surveys faced criticism for the insufficient time for feedback, development, and implementation of workplace health promotion efforts until it was time for the next employee survey. Also, the employee surveys were criticized for focusing mainly on negative outcomes, rather than on positive ones. This created challenges in interpreting and taking actions based on the results [26].

## Discussion

Out of 1550 potential articles, only two provided information on whether employee surveys are effective tools for promoting health initiatives in the workplace [25, 26]. In the interview study by R Larsson, I Åkerlind and H Sandmark [26], managers described the employee survey as an important tool in managing workplace health promoting programs. The employee surveys were described as being used as a way to solve problems and to map health and working conditions, in order to improve employee health through action plans based on data from the surveys. TM Schult, SK Schmunk and ER Awosika [25] used a wellness survey alongside the employee survey, evaluating effects of a workplace health promoting initiative, indicating that employee surveys, along with wellness questions, could be used to improve employee health, which is in line with the results by R Larsson, I Åkerlind and H Sandmark [26]. Additionally, none of the eligible studies applied a theory, model or framework to promote behavior change among employees. This may be explained by the fact that only the study by TM Schult, SK Schmunk and ER Awosika [25] aimed to influence behavior change of employees. This demonstrates that employee surveys may be underutilized in health promotion initiatives to change the workplace environment and support behavior change. The workplace is a complex social environment including legislations and cultural norms, as well as physical factors such as ergonomics, building design, and the natural environment, all of which influence employee health behaviors [33]. To design effective employee surveys and interventions that improve both employee health and performance, organizations need theory-informed guidance. Future research should therefore integrate relevant theoretical frameworks when designing employee surveys, interpreting the results and implementing actions to create change. One such framework is perceived organizational support, which reflects the extent to which employees believe their organization values their well-being and contributions [34], for example in relation to leadership, employee engagement, and health and safety that are frequent areas in employee surveys. Moreover, broad frameworks, such as the Social Ecological Model [35] and the Behavior Change Wheel [36], offer more guided approaches than models that focus solely on individual behavior change [35]. Such frameworks may enable managers and human resources professionals to understand important areas for employee behavior change, inform the construction of employee surveys as well as identify interventions that acknowledge both socio-cultural and physical dimensions of the workplace [33].

An important function of employee surveys is the follow-up of the results and the implementation of change based on the results, ideally through relevant action plans at the workplace [1, 4]. However, few eligible studies address this, despite using employee survey data. Only three studies discuss implementation of survey results: L Jarman, A Martin, A Venn, P Otahal, R Taylor, B Teale and K Sanderson [30] reported that the studied organization implemented a strategy for solving the issues covered in the study; R Larsson, I Åkerlind and H Sandmark [26] reported that the managers criticized the employee survey process as suboptimal for work health initiatives; and L Lindert, S Zeike, K-EA Choi and H Pfaff [24] considered the employee survey as an appropriate tool for workplace health promotion. A similar pattern emerges regarding the analysis of differences by gender, level of education and other demographic variables of the employees as well as workplace positions. Five out of the eight studies discuss or analyze the results with regards to these aspects [24, 28–31], though the reporting was generally limited, especially in the two studies primarily focusing on physical health and healthy lifestyle [25, 31].

Using employee surveys to promote healthy workplaces benefits both employee’s health and the organizational outcomes [9–12]. The employee survey should capture not only work-related issues, e.g., leadership or work tasks, but also health-related factors, e.g., physical activity, fruit or vegetable consumption, sleep or recovery. Current research on employee surveys rarely applies this health promoting perspective [2, 4–6, 37, 38]. These surveys can be beneficial for identifying problems that need to be addressed, and evaluating solutions to these problems, making them vital tools in the problem-solving loop process, as implied by R Larsson, I Åkerlind and H Sandmark [26].

In the new Corporate Sustainability Reporting Directive [17], a description of the organization’s impact on social sustainability will be mandatory for companies in the European Union. Thus, employee surveys linked to health promoting initiatives at the workplace will be a way to highlight improvements and be a vital part in the sustainability report. This highlights the need for evidence-based methods and the use of health-related key performance indicators (KPI: s) to evaluate how effectively the organization is achieving its objectives.

Using employee surveys for health promotion may encounter the same problems as for regular employee surveys, for example, problems with negative attitudes towards the survey [2], response biases like social desirability and non-response [2, 6], or difficulties interpreting or managing the results [26, 37, 38]. It is important to reduce and prevent problems like these by planning ahead before the survey process begins. For instance, to reduce response biases in employee surveys, NL Keiser and SC Payne [6] suggests using a third party, such as an external consultancy, to perform the survey instead of the organization itself. This approach ensures that individual responses remain anonymous to the employer, which may encourage more honest answers, reducing the risk of social desirability. NL Keiser and SC Payne [6] also recommends rephrasing survey questions from an individual to a group perspective, shifting the focus from individual experiences to the wider system. These solutions could easily be integrated in a tailored employee survey designed specifically for a specific workplace. Tailoring the survey is recommended to increase employee participation and support the development of useful action plans based on the results [37]. Once action plans have been implemented, it is important to clearly communicate the changes to employees. Doing so can increase participation in upcoming surveys, as employees are more likely to see the survey as useful when they observe that their feedback leads to improvements at the workplace [2].

### Strengths and weaknesses

The scoping review used a thorough and transparent process for identifying relevant research articles and the search strategy was developed with the help of librarians from Mälardalen University to ensure a broad search strategy. Five databases were used as sources for articles and the reference lists of all eight articles eligible for the review were screened for additional articles. The full review process, from title and abstract screening to data charting, was made by two independent reviewers who held recurrent meetings to discuss and resolve conflicts and share insights during the process. To better manage articles and data, the softwares Covidence and Excel were used, enabling the reviewers to easily share and ensure coherent data was collected. Since this is a scoping review, the reviewers ensured to be transparent and generous with the articles collected and deemed eligible, always consulting with the last author to validate inclusion of potential studies. Consequently, all articles deemed eligible have been reviewed by at least three of the authors.

For the search strategy, the authors used every known synonym for “employee survey.” However, there may have been additional terms not included in this review due to unknown terminology when creating the search strategy, potentially resulting in missed articles. For example, an organization may regularly conduct a health risk assessment survey for the same purpose as an employee survey, but with a more specific health focus than this review aimed to identify. Furthermore, the review excluded all gray literature which is generally encouraged to be included in scoping reviews [19]. Limiting the search strategy to the last 10 years might also affect the inclusion of studies, since studies older than 10 years still might have been eligible for inclusion. Lastly, only eight studies were deemed eligible for inclusion in the study. This small sample size, in combination with their heterogeneous characteristics, makes it difficult to generalize the findings in accordance with workplace, types of business, country or other characteristics. Thus, this scoping review highlights a significant knowledge gap regarding the use of employee surveys for health promotion at work. Future research should therefore focus on what survey dimensions are suitable for capturing health and lifestyle among employees, how organizations translate survey findings into effective interventions and how these are implemented and monitored. This should be done in longitudinal studies with multiple assessments, stretching over several years, as well as in short term intervention studies to evaluate specific actions. Moreover, future reviews may expand the search strategy to also include gray literature or other sources, for example, from human resources professionals and managers, to gain insights on whether and how employe surveys are being used to promote employee health.

Some large-scale employee surveys were identified in the search strategy, initially appearing eligible but ultimately excluded. One example is the BIBB/BAuA employment survey of the working population on qualification and working conditions in Germany [39]. Although some studies used this survey to investigate topics relevant for eligibility, for example shoulder pain [40], the data were collected at a national level and thus were not linked to any specific companies or organizations. Consequently, national population surveys did not support the development of action plans or targeted health promotion initiatives within individual workplaces and were therefore excluded from this study. In contrast, employee surveys conducted within specific workplaces provide data tailored to the unique conditions, risks, and needs of each organizational setting in line with the aim of this study. This makes employee surveys particularly valuable for targeted workplace health promotion and organizational development. Employee and workplace specific insights can support human resources professionals and managers with improving employee survey design and processes, identifying ill health at the workplace, and developing effective health promoting interventions for employees.

### Ethical aspects

Including workplace health promotion aspects in employee surveys may raise ethical concerns among employees, particularly regarding invasion of privacy. Participants in the study by R Larsson, I Åkerlind and H Sandmark [26] discuss whether it is morally correct to influence the employees’ health behavior, since it interferes with private life. Therefore, it is important to implement workplace health questions in a respectful manner, emphasizing voluntary participation. Future research should examine ethical implications related to vulnerable groups, individuals with obesity, alcoholism, smokers or unhealthy behaviors that might be associated with stigmatization when filling out the employee survey. Moreover, it is also important to explore whether there might be areas of conflict between economic gain and employee health [41, 42].

### Future research

The result of this scoping review indicates that health-related questions are scarce in the research of employee surveys. Thus, future research should explore if employee surveys can be linked to promoting and following employee health and lifestyle over time at work. Therefore, it is important to examine what health-related questions are relevant to include in employee surveys to promote better health and lifestyle among employees, including what questions or topics employees are comfortable answering and what areas are realistic for change. Mixed-method intervention studies may be useful to both test and understand how health-related questions in employee surveys are received by employees and used by management. Lastly, it is important to consider what health-related questions are ethically appropriate to include, and how to use the data responsively without stigmatizing unhealthy groups. Additionally, it is important to investigate the balance between prioritizing business objectives and employee health to find a win-win situation.

## Conclusions

The role of physical health and healthy lifestyles seems to appear to be outside the scope of employee surveys as portrayed in research. This highlights a significant knowledge gap in the scientific literature, since the employee survey is one of the most utilized and widespread tools for organizational development, and that human capital is the most valuable resource of an organization. Also, employee surveys offer tailored data for a specific workplace and its employees, valuable for health promotion and organizational development, which large-scale population-based surveys miss. Some of the key questions that are highlighted in this review and need to be explored in further studies includes identifying relevant health and lifestyle-related questions, how organizations translate survey findings into effective health interventions and how these actions are implemented and monitored using theories for behavior change. Moreover, future research should explore the process of using employee surveys for identifying health related problems, implementing health promotion solutions, follow-up improvements within the organization, and thereby creating a workplace health promoting loop process. Thus, both intervention studies and longitudinal studies with multiple assessments are needed to investigate how to improve and maintain good employee health over time.

## Supplementary Information

Additional file 1.docx: Description of eligible studies. The data describes the eligible studies used for this review.

## Data Availability

The data and material supporting the conclusions of this article is included within the article and additional file 1.
