# Supplementary material for: The role of employee surveys to promote physical health and healthy lifestyles at the workplace: a scoping review
Source: BMC Public Health. 2025 Sep 24;25:3054. doi: 10.1186/s12889-025-24716-7 (PMC12459028; doi:10.1186/s12889-025-24716-7)
Supplement: Supplementary file 1 — Additional file 1.docx: Description of eligible studies. The data describes the eligible studies used for this review. [file 12889_2025_24716_MOESM1_ESM.docx]

Description of eligible studies.

| **Author, year** | **Study design** | **Aim** | **Sample and size** | **Occupational setting** | **Main focus** | **Health outcomes** | **The role of the employee survey** |
| --- | --- | --- | --- | --- | --- | --- | --- |
| Hämmig, 2017 | Cross-sectional | To explore whether and in what way social support from different sources and domains makes an additional or different and independent contribution to various health and work-related outcomes | Employees at four different companies,  N = 5,877 | Insurance, banking, transportation & healthcare | Social support | General health, musculoskeletal disorders, stress, burnout | Data source for investigating relationships |
| Jarman, Martin, Venn, Otahal, Taylor, Teale & Sanderson, 2014 | Cross-sectional | To compare the self-reported prevalence of psychological distress measured with a brief screening-tool with that of an employer survey; determine whether prevalence differed to normative population data; and investigate and classify the socio-demographic, health and work correlates of self-reported high psychological distress | Employees at the Tasmanian state service, Workforce survey (H@W 2009) –  N = 7,715, researcher survey (PH@W) –  N = 3,406 | The Tasmanian state service (e.g., health, education and environment) | Psychological distress | General physical health, smoking habits, fruit and vegetables intake, BMI, alcohol intake, physical activity, psychosocial factors | Data source for investigating relationships and comparing results between the employee survey, the researcher survey and population norms |
| Larsson, Åkerlind & Sandmark, 2016 | Interview study | To explore how workplace health promotion is managed and incorporated into the general management system in two large Swedish municipal organizations | Senior managers,  N = 14 | Childcare and education, elderly and social care, traffic and urban planning, environment, human resources, municipal district administration | Managing workplace health promotion |  | A tool managers use for assessing workplace needs and promoting employee health |
| Lindert, Choi, Pfaff & Zeike, 2023 | Cross-sectional | To investigate the impact of organizational health literacy and health supporting leadership on the relationship between individual health literacy and employees’ psychological wellbeing in a big German company based in the financial sector | Employees at a large company, N = 2,555 | Financial | Individual and organizational health literacy, psychological wellbeing, health supporting leadership |  | Data source for investigating relationships |
| Lindert, Zeike, Choi & Pfaff, 2023 | Longitudinal | (Hypothesis 1) The difference in perceived transformational leadership over time significantly impacts psychological wellbeing. (Hypothesis 2) The difference in psychological wellbeing over time significantly impacts the leadership score at t1 | Employees at a company,  N = 127 | Lighting industry | Transformational leadership, psychological wellbeing, social capital |  | Data source for investigating longitudinal relationships |
| Loekke, 2016 | Cross-sectional | To investigate the relationship between workplace social capital and health and job-related outcomes in a large Danish municipality | Employees in a Danish municipality,  N = 4,162 | Municipality in public sector | Social capital | General health, psychological distress, social support | Data source for investigating relationships |
| Piranveyseh, Motamedzade, Osatuke, Mohammadfam, Moghimbeigi, Soltanzadeh & Mohammadi, 2016 | Cross-sectional | To investigate the relationship between organizational and personal (individual) factors with the prevalence of musculoskeletal disorders (MSDs) in office workers of the Iranian Gas Transmission Company | Office employees at a company,  N = 143 | Gas transmission | Musculoskeletal disorders, organizational climate | BMI | Data source for investigating relationships |
| Schult, Schmunk & Awosika, 2016 | Quasi-experimental | To describe a comprehensive approach to decrease physical inactivity at the VHA employee population | Employees at the VHA workplace,  t^1^ – N = 29,834,  t^2^ – N = 86,257 | Healthcare | Physical inactivity |  | Data source for assessing workplace health promotion interventions at two time-points, pre-post survey measure |
